# Enhancing Blood Circulation With Epsilon‐Near‐Zero (ENZ) Materials via the Far‐Infrared Window of Human Skin

**DOI:** 10.1002/advs.75938

**Published:** 2026-06-15

**Authors:** Wen‐Teng Yao, Shan‐Chiao Yang, Ming‐Feng Tsai, Hsuen‐Li Chen

**Affiliations:** ^1^ Department of Materials Science and Engineering National Taiwan University Taipei Taiwan; ^2^ Department of Medicine MacKay Medical University Taipei Taiwan; ^3^ Division of Plastic Surgery Department of Surgery MacKay Memorial Hospital Taipei Taiwan; ^4^ Center of Atomic Initiative for New Materials National Taiwan University Taipei Taiwan

**Keywords:** blood flow, epsilon‐near‐zero (ENZ) material, far‐infrared (FIR) skin window, human skin window, laser speckle contrast imaging (LSCI), selective‐band emitter

## Abstract

Although the near‐infrared (NIR) window is widely recognized for its deep tissue applications, the far‐infrared (FIR) region, centered near 10 µm, remains largely underexplored. Human skin exhibits a relative absorption minimum in this range, suggesting the potential to deliver thermal energy more deeply while avoiding superficial overheating. Epsilon‐near‐zero (ENZ) materials provide this capability as their optical resonances confine thermal emission to specific wavelengths. Silicon carbide (SiC) exhibits an ENZ resonance near 10 µm, which aligns directly with the FIR skin window. Here, we report the first systematic investigation of wavelength‐matched FIR therapy, comparing SiC with the conventional broadband emitter, graphite. A thermally controlled within‐subject study of 25 volunteers (50 hands) simultaneously monitored real‐time blood perfusion and cardiovascular parameters. After 20 min of irradiation, SiC increased cutaneous blood flow by an average of 30.2% relative to baseline, significantly exceeding the 18.6% increase observed with graphite. These enhancements occurred without changes in blood pressure or heart rate, and skin surface temperature remained below 36°C. These findings establish wavelength‐matched FIR therapy as an effective and thermally safe approach for enhancing peripheral circulation without eliciting systemic effects.

## Graphical abstract

Epsilon‐near‐zero (ENZ) silicon carbide enables a selective far‐infrared emitter spectrally aligned with the human skin window around 10 µm for wavelength‐matched thermal therapy. In 25 within‐subject trials (50 hands) monitored by laser speckle contrast imaging, SiC increases blood perfusion by 30.2% versus a broadband graphite emitter while maintaining stable blood pressure and heart rate and keeping skin temperature below 36°C.

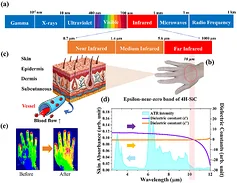

## 1 Introduction

The wound healing process comprises four distinct phases: hemostasis, inflammation, proliferation, and remodeling. Each phase requires adequate blood circulation to deliver nutrients and oxygen to the skin and underlying tissues. Maintaining optimal blood flow is essential to support these healing stages and to ensure that vital nutrients reach the affected tissues. Conditions such as diabetes mellitus, peripheral arterial occlusive disease, and acute trauma can impair blood circulation in the skin, leading to non‐healing wounds [1]. A variety of treatments have been developed to improve blood circulation in the extremities. According to the 2016 American Heart Association (AHA) and American College of Cardiology (ACC) guidelines for lower extremity peripheral artery disease, antiplatelet therapy, such as oral medications, aspirin, or clopidogrel, is the recommended first‐line treatment. Endovascular interventions for claudication include balloon dilation (angioplasty), stents, and atherectomy. Surgical revascularization, commonly referred to as bypass surgery, serves as the final recourse for critical limb ischemia [2]. Infrared radiation (IR) therapy has emerged as a noninvasive approach with potential benefits for wound healing. Far‐infrared radiation (FIR), in particular, has been shown to exert both thermal and nonthermal biological effects that may enhance blood circulation and promote healing processes [3].

IR radiation is divided into three spectral regions: near‐infrared (NIR, 0.7–1.4 µm), mid‐infrared (MIR, 1.4–5.6 µm), and FIR (5.6–1000 µm). Longer‐wavelength light, particularly in the IR range, penetrates deeper into biological tissues than visible light, which reaches only the skin's outermost layers. Specifically, human skin exhibits an NIR optical window between 700 and 1400 nm, which allows for deeper tissue penetration, reduced scattering, and minimal autofluorescence, making it an ideal optical range for light‐based therapeutic applications [4, 5]. This NIR wavelength range also enhances the signal‐to‐noise ratio, which is critical for both imaging and therapeutic applications.

Several fluorescent nanomaterials, including carbon nanotubes (CNTs), quantum dots (QDs), and polymer nanoparticles (NPs), that absorb and emit light within the NIR range are employed for high‐resolution deep tissue imaging. Hollow gold nanoparticles (HGNs) possess unique optical properties and low toxicity, making them well‐suited for biomedical applications [6]. Because of their strong absorption in the first NIR window (700–900 nm), HGNs function as nano‐heaters in photothermal therapy. Light in the second NIR window (1000–1700 nm) offers several advantages, including lower absorption by biological molecules such as hemoglobin, reduced scattering, and diminished background fluorescence, thereby enhancing imaging depth and quality. NIR‐induced photothermal modulation offers multimodal benefits, including protection against ventricular arrhythmias during myocardial ischemia‐reperfusion [7]. NIR‐based cancer imaging and therapy have several advantages, including non‐invasiveness, deep‐tissue penetration, and molecular specificity [8]. Photothermal therapy is a promising technique that employs photothermal agents to convert NIR radiation into heat, thereby eliminating bacteria and promoting tissue regeneration [9].

Furthermore, FIR radiation has been investigated for its biological effects, particularly its role in enhancing blood circulation and promoting tissue repair. Although FIR is known to penetrate tissues with minimal absorption, the biological effects of specific FIR wavelengths remain underexplored. Attenuated total reflectance Fourier‐transform infrared (ATR‐FTIR) spectroscopy of human skin [10] reveals several characteristic absorption bands: the X─H stretching region (2.5–4 µm) [11]; the triple‐bond region (4–5 µm) corresponding to C≡C and C≡N vibrations [12]; the double‐bond region (5–6.7 µm) associated with absorptions from C═C, C═O, and C═N bonds; and the fingerprint region (6.7–16.7 µm) [13, 14]. Interestingly, within this fingerprint region, a weak absorption band consistently appears near 10 µm. This subtle trough indicates a potential FIR transmission window, capable of reaching subdermal vasculature without overheating the skin surface. However, this spectral region has not been explored in the context of selective thermal therapy.

Several studies have demonstrated the biological benefits of FIR radiation. Lin et al. investigated hemodialysis patients who underwent FIR therapy for one year. The FIR emitter utilized electrically heated ceramic plates to generate electromagnetic waves. The results indicated that FIR therapy improved vascular access flow, with nonthermal and long‐term effects contributing critically to the survival of arteriovenous fistulae in hemodialysis patients [15]. Yu et al. found that FIR exposure increased skin microcirculation in rats, attributed to the release of nitric oxide (NO), a signaling molecule that induces vasodilation and enhances blood flow. A 45‐min FIR treatment significantly stimulated skin blood flow, with effects persisting for up to 60 min [16]. Hsu et al. demonstrated that FIR exposure enhanced the migration of rat renal tubular epithelial cells by improving mitochondrial function. FIR radiation has also been shown to enhance mitochondrial function, a key driver of tissue repair and regeneration. An optimal therapeutic response was observed at an irradiation distance of 3–4 cm from the skin surface and a duration of approximately 30 min [17].

In clinical practice, FIR therapy typically relies on broadband thermal sources such as IR lamps, FIR saunas, and commercial ceramic emitters [18]. These devices are widely accessible and safe but lack spectral selectivity, limiting their ability to deliver energy efficiently to specific IR wavelength ranges, particularly those corresponding to optical transmission windows in human skin. As a result, their effectiveness in targeted thermal therapy and biological modulation remains limited. Although mid‐ and FIR lasers, such as quantum cascade lasers (QCLs), provide precise control over output wavelengths, their high cost and system complexity render them impractical for routine clinical applications [19]. These limitations have spurred growing interest in developing low‐cost, spectrally selective FIR emitters that efficiently target biologically relevant IR wavelengths for more effective, mechanism‐driven photothermal treatment.

Typical FIR‐emitting ceramics include metal oxides such as silicon dioxide (SiO_2_), aluminum oxide (Al_2_O_3_), and titanium dioxide (TiO_2_) [20]. These ceramic materials have shown potential for thermal management applications, as well as in functional textiles and wearable devices that provide a range of health benefits [21]. In many commercial products, fabrics infused with FIR‐emitting ceramic nanoparticles are woven into garments or wraps, where the wearer's body heat passively generates FIR radiation. This radiation is believed to enhance comfort, promote local blood flow, and support overall circulatory function [22]. Graphite is another commonly used FIR‐emitting material in thermal systems. As a semimetallic allotrope of carbon, graphite exhibits high thermal conductivity and can be readily processed into thin films or coatings [23]. However, the emission spectrum of graphite is inherently broadband, lacking the spectral selectivity required for precise alignment with biological optical windows. Furthermore, the biological effects of FIR radiation are insufficiently characterized, with key parameters such as power density, distance, frequency, and radiation duration still under investigation.

In recent years, epsilon‐near‐zero (ENZ) materials have attracted considerable interest for their ability to achieve high thermal emissivity at specific IR wavelengths [24]. When the real part of the permittivity approaches zero, ENZ media exhibit uniform phase distribution [25], strong field enhancement [26], and unusual wave phenomena such as supercoupling [27] and phase tunnelling [28]. Nonlinear ENZ nanodevices exhibiting near‐vanishing permittivity have also been demonstrated [29, 30]. Recent studies have shown that intrinsic phonon resonances in polar ENZ materials can confine thermal emission into narrow spectral bands [31]. This spectral selectivity has been widely utilized in mid‐ and FIR technologies, including thermophotovoltaics [32] and radiative cooling [33], where precise control of the emission wavelength is essential for improving energy conversion efficiency and reducing thermal load. Nevertheless, no prior work has reported the use of ENZ‐based emitters for biomedical selective thermal therapy, where matching the emission spectrum to the biological transparency window could enable deeper tissue penetration while minimizing unintended heating of surrounding areas. Addressing this spectral window could open new opportunities for noninvasive and highly targeted thermal therapy.

Silicon carbide (SiC) is a polar dielectric material characterized by its strong optical phonon resonances in the IR spectral range, which give rise to a well‐defined Reststrahlen band within the FIR region [34]. These optical characteristics enable SiC to selectively emit thermal radiation, potentially enhancing energy efficiency in IR‐based applications [35]. Moreover, the covalently bonded crystal structure of SiC imparts exceptional thermal stability [36], chemical resistance [37], and mechanical strength [38], making it advantageous for both harsh‐environment operation and biocompatible therapeutic platforms, and it can also serve as a functional reinforcing filler [39]. Despite these attributes, the use of SiC‐based ENZ structures for targeted FIR biomedical applications remains scarcely explored in the literature [40].

This study employed a single‐crystal SiC wafer as a selective‐wavelength emitter for FIR thermal therapy on human hands and compared its performance with that of graphite under identical heating temperatures and irradiation durations. Changes in cutaneous blood flow were evaluated using laser speckle contrast imaging (LSCI). Vital signs, including blood pressure, heart rate, and oxygen saturation, were recorded before and after irradiation. We hypothesized that SiC‐based FIR emitters represent a practical approach for enhancing blood flow, promoting local tissue perfusion, and providing a more efficient alternative to conventional ceramic emitters.

## 2 Results and Discussion

### 2.1 Characterization of Infrared Radiation and Optical Window in Human Skin

**FIGURE 1:**
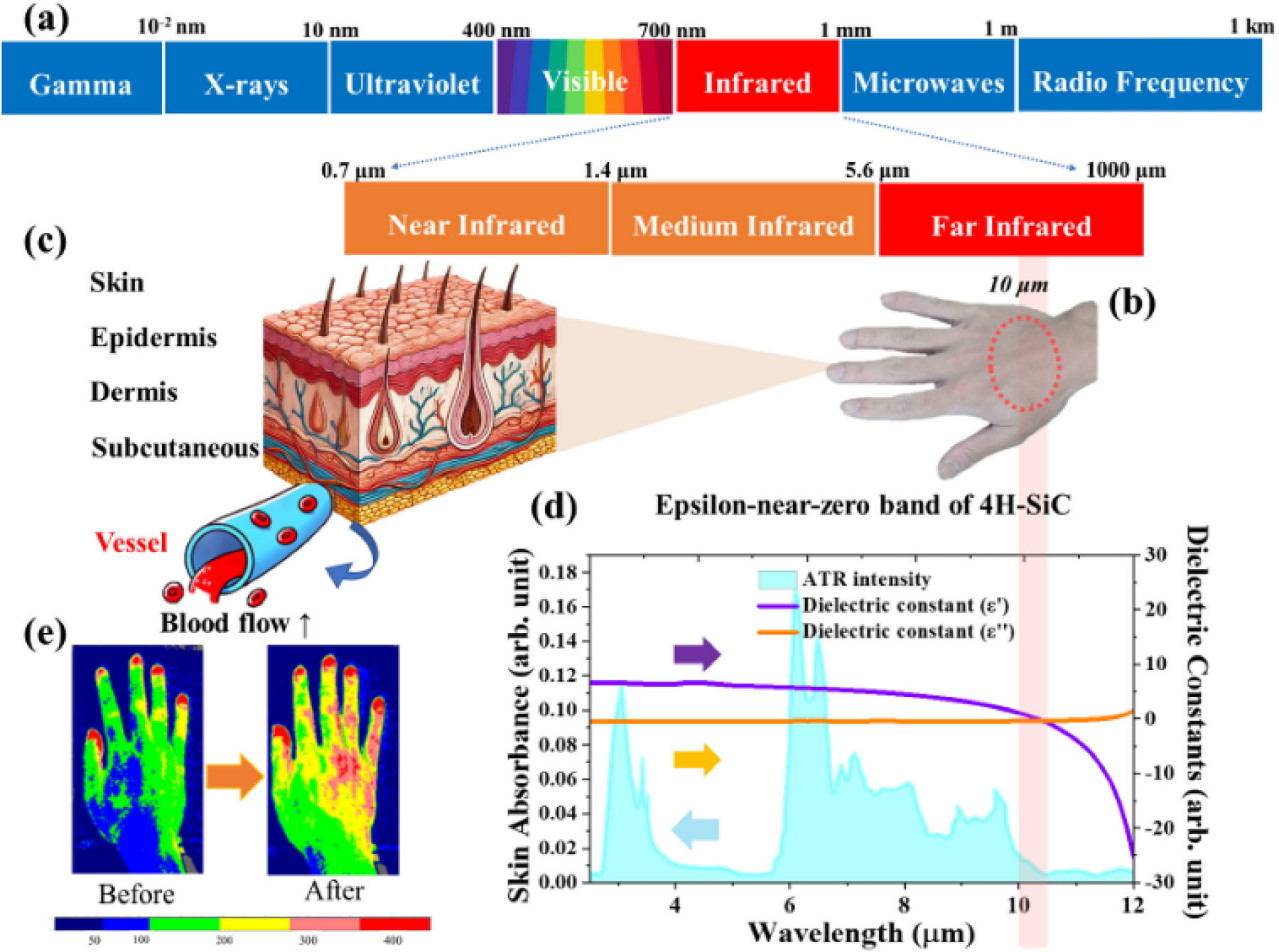
Overview of FIR‐skin interaction and ENZ material matching. (a) Schematic illustration of the electromagnetic spectrum highlighting the skin's optical window. (b) Depiction of FIR light penetration through the epidermis layer into subdermal blood vessels, potentially enhancing blood flow. (c) Anatomical structure of the skin, composed of the epidermis, dermis, and subcutaneous layer, where dermal and hypodermal blood vessels contribute to skin circulation. (d) Comparison between the dielectric constant of SiC and the FIR skin window. The shaded blue background represents the ATR‐FTIR spectrum of human skin, while the overlaid curves indicate the real (ε’) and imaginary (ε″) parts of SiC's dielectric constant, demonstrating its ENZ behavior near 10 µm. (e) Laser speckle contrast imaging (LSCI) of the hand before and after FIR irradiation, showing increased perfusion post‐treatment (left: before; right: after).

The electromagnetic spectrum encompasses radio waves (wavelengths from 1 m to several km), microwaves (1 mm to 1 m), infrared radiation (700 nm to 1 mm), visible light (400 nm to 700 nm), ultraviolet (UV) radiation (10 to 400 nm), X‐rays (0.01 to 10 nm), and gamma rays (wavelengths less than 0.01 nm). As shown in Figure 1a, infrared radiation is divided into near‐infrared (NIR, 0.7–1.4 µm), middle‐infrared (MIR, 1.4–5.6 µm), and far‐infrared (FIR, 5.6–1000 µm) regions. To explore the biological potential of FIR radiation, we first identified a selective low‐absorption window near 10 µm in human skin using attenuated total reflectance Fourier‐transform infrared (ATR‐FTIR) spectroscopy. As shown in Figure 1b, human skin exhibits characteristic vibrational absorption bands within the 2–12 µm range, corresponding to molecular components such as proteins, lipids, and nucleic acids. Notably, an optical window in human skin was observed near 10 µm, suggesting a promising wavelength band for FIR‐based thermal penetration. In this study, we employed an FIR‐emitting material with strong, spectrally selective emission characteristics in this wavelength range to examine its effects on skin microcirculation.

The anatomical structure of the skin supports this approach. It is the largest organ of the human body. The skin is composed of three primary layers: epidermis, dermis, and hypodermis (subcutaneous layer). The epidermis forms the outermost layer and serves as a barrier between the body and the external environment. It protects against pathogens, regulates water loss, and contributes to skin regeneration. The dermis, located beneath the epidermis, is rich in collagen and elastin, which provide the skin with strength and flexibility. The hypodermis is the deepest layer, consisting primarily of fat and connective tissue. As shown in Figure 1c, blood vessels are distributed throughout the dermis and hypodermis, where blood circulation regulates temperature and supports oxygen and nutrient delivery, healing, and immunity. FIR radiation can penetrate these layers (up to 4 cm beneath the skin) and can effectively modulate circulatory function [41].

We hypothesized that matching the FIR emission spectrum to this low‐absorption window would selectively enhance energy deposition in deeper skin layers, thereby improving blood circulation. Therefore, we employed SiC, an epsilon‐near‐zero (ENZ) material with a strong emission band around 10 µm, as a selective thermal emitter for biomedical applications. As shown in Figure 1d, changes in blood flow in the human hand were observed using laser speckle contrast imaging (LSCI). This measurement is illustrated in Figure 1e.

**FIGURE 2:**
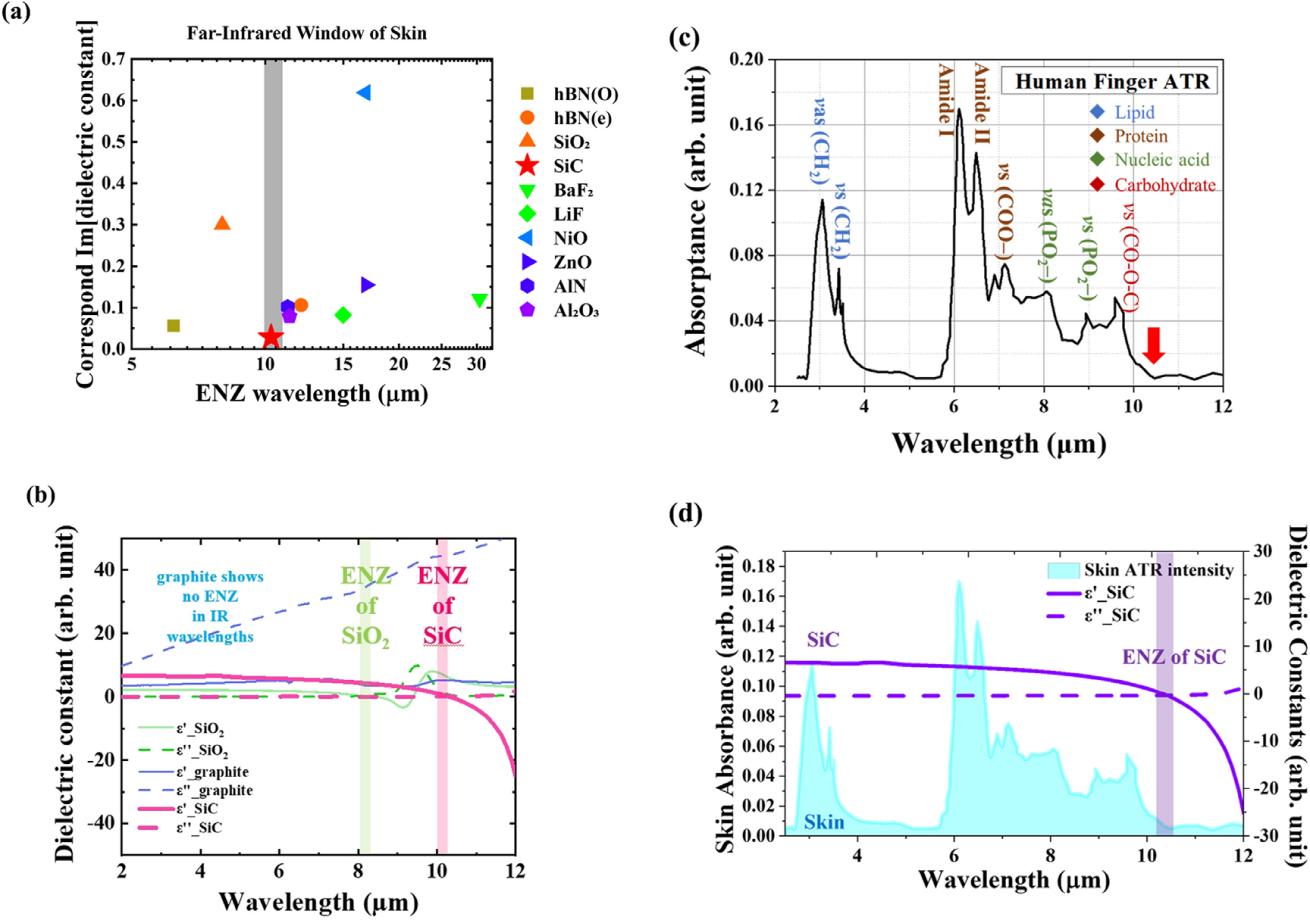
Optical properties of FIR emitters and spectral comparison with human skin. (a) ENZ wavelengths and corresponding Im[ε] values for various polar dielectric materials. Lower Im[ε] indicates reduced optical damping at the ENZ point, favorable for efficient FIR emission. The gray band highlights the FIR spectral region relevant to skin transmission. (b) Real (ε′, solid lines) and imaginary (ε″, dashed lines) parts of the dielectric constants of SiC, SiO_2_, and graphite. SiC and SiO_2_ exhibit pronounced ENZ points at approximately 10.3 and 8.1 µm, respectively, whereas graphite shows a broadband low ε′ region but without a distinct ENZ feature. (c) ATR‐FTIR spectrum of human finger skin in the 2–12 µm range, showing characteristic absorption peaks associated with biomolecular vibration of lipids, proteins, carbohydrates, and nucleic acids. (d) Spectral alignment of SiC's ENZ behavior with the low‐absorption window of human skin near 10 µm, confirming its role as a targeted FIR emitter for biomedical applications.

To further evaluate suitable materials for FIR thermal modulation, we analyzed the dielectric properties of several candidate emitters. Figure 2a shows the IR wavelengths at which representative polar dielectric materials exhibit near‐zero real permittivity (ε’≈ 0), along with their corresponding imaginary components (Im[ε]). Among these materials, SiC shows a very low Im[ε] near 10.3 µm, allowing minimal optical damping and promoting strong phonon‐polariton resonances. These resonances enable spectrally confined thermal emission within the FIR range [42]. Notably, this emission wavelength closely aligns with the skin optical window near 10 µm, as shown in Figure 1.

### 2.2 Dielectric Properties and ENZ Behavior of FIR Emitters

Figure 2b compares the dielectric constants of SiC, SiO_2_, and graphite across the mid‐ and far‐infrared range (2–12 µm), illustrating how fundamentally different classes of materials exhibit distinct infrared responses. SiC and SiO_2_, as typical polar dielectrics, exhibit ENZ behavior near 10.3 and 8.1 µm, respectively, where the real part of the permittivity approaches zero and strong phonon–polariton interactions cause pronounced dispersion in both the real and imaginary components. However, graphite shows a broadband low ε’ region but without a distinct ENZ feature. In contrast, graphite [43] displays a weakly dispersive and nearly featureless dielectric response, consistent with its conductive nature, which supports broadband thermal emission without wavelength specificity. This comparison underscores the distinct optical mechanisms of polar and conductive materials, establishing the foundation for subsequent evaluation of SiC as a wavelength‐matched FIR emitter.

To establish the biological relevance, Figure 2c presents the ATR‐FTIR spectrum of human finger skin in the 2 to 12 µm range. Distinct absorption peaks appear near 3.0, 6.0, and 6.5 µm, corresponding to vibrational modes of proteins, lipids, and water. An optical window in the skin is observed near 10 µm, where absorption of the FIR radiation is minimal.

Building on this, Figure 2d overlays the dielectric function of SiC with the skin absorption profile. SiC exhibits ENZ behavior near the wavelength of 10 µm, where its permittivity approaches zero and strong phonon–polariton interactions align with the skin optical window. This spectral alignment provides a compelling physical rationale. Unlike broadband emitters such as graphite, SiC has the potential to couple energy efficiently into deeper skin layers while minimizing superficial heating. This rationale motivated the subsequent physiological experiments, where SiC and graphite were directly compared under controlled conditions.

### 2.3 Physiological Response of Human Skin to FIR Irradiation: A Controlled Comparison

**FIGURE 3:**
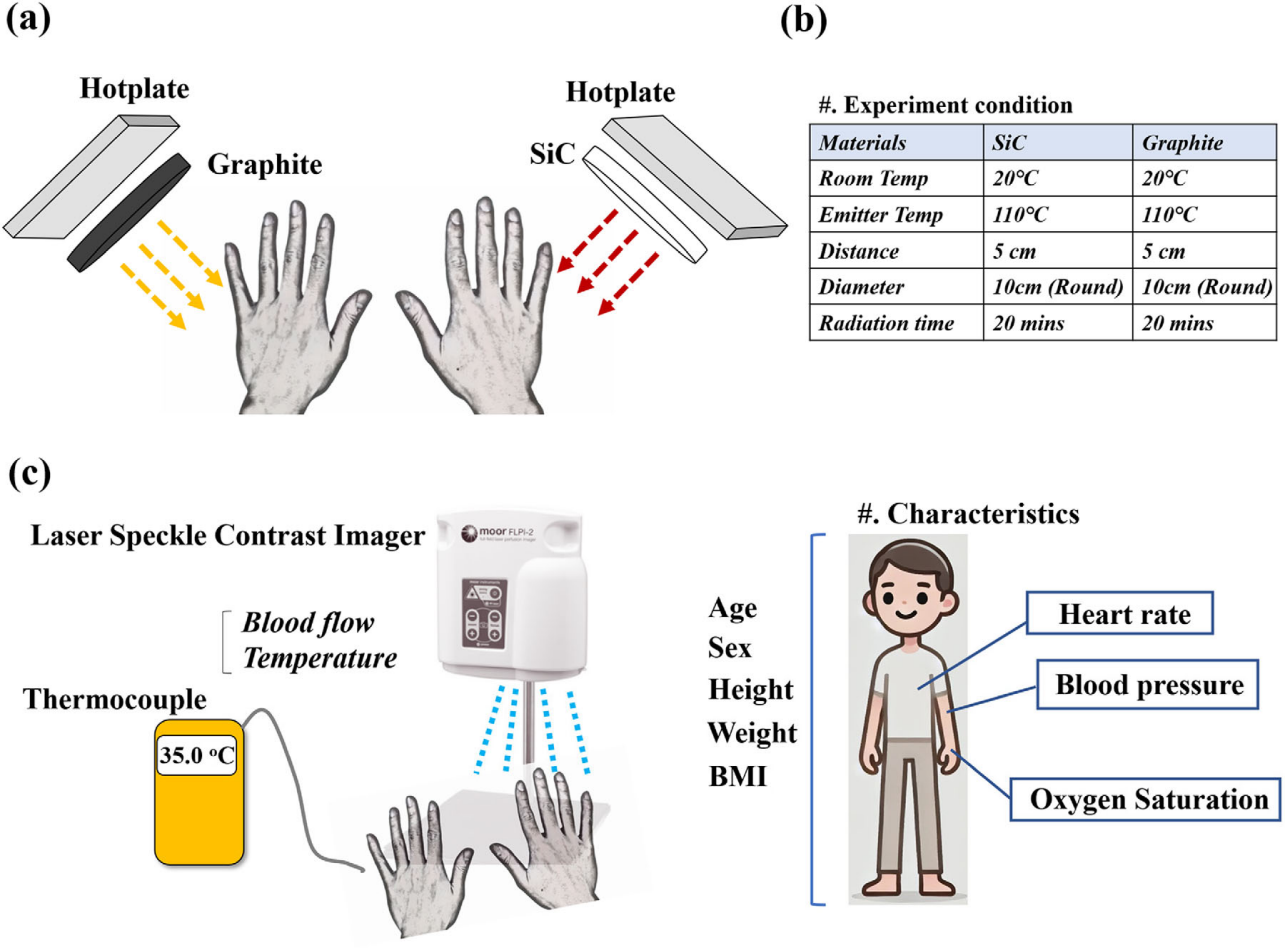
Experimental setup for FIR irradiation and physiological monitoring. (a) Schematic of the experimental configuration, where the left hand was irradiated using a graphite FIR emitter mounted on a hotplate, and the right hand was exposed to a SiC emitter under identical thermal conditions. (b) Standardized hand positioning ensured symmetric exposure geometry and thermal equivalence. (c) Laser speckle contrast imaging (LSCI) was employed to monitor real‐time blood flow dynamics, while thermocouples recorded skin surface temperature. Concurrently, physiological parameters, including blood pressure, age, sex, height, weight, body mass index (BMI), heart rate, and oxygen saturation, were recorded to ensure safety and enable correlation analysis.

Building on these spectral properties, we designed a controlled experimental framework to evaluate the physiological response of human skin to selective FIR irradiation. The overall experimental design is shown in Figure 3a–c. In this within‐subject configuration (Figure 3a), both hands of each participant were irradiated simultaneously under identical thermal conditions. One hand was exposed to a SiC emitter exhibiting ENZ resonance, while the contralateral hand was exposed to a broadband graphite emitter. This setup enabled a direct, controlled comparison of the circulatory effects of selective and broadband FIR emission.

The emitters (Figure 3b) were mounted on a thermostatically controlled hotplate and heated to 110°C (383 K), a temperature sufficient to generate a measurable FIR output. The emitters were positioned 5 cm above the dorsal surface of the hands, and irradiation was maintained for 20 min at an ambient temperature of 20°C. Physiological monitoring (Figure 3c) was performed using laser speckle contrast imaging (LSCI) to capture dynamic changes in blood perfusion across the dorsal hand in real time. The LSCI system was positioned 25 cm above the irradiated area and operated at a temporal resolution of 50 ms, enabling quantitative assessment of cutaneous circulation. Thermocouples were simultaneously attached to the skin surface to monitor temperature and ensure thermal safety throughout the experiment.

Following this setup, 25 healthy volunteers were enrolled, providing 50 hands for irradiation trials. Participants had a mean age of 50.8 years (range: 28–77), comprising 18 females and 7 males. No participants had major comorbidities, including diabetes mellitus, chronic heart disease, or chronic kidney disease. Baseline anthropometric parameters, including height, weight, and body mass index (BMI), are summarized in Table 1.

**TABLE 1:** Baseline demographic and anthropometric characteristics of study participants.

Characteristic (*n* = 25) Values		Range
Age (Years)	50.8	28–77
Sex (Male/ Female)	7 M/18F	—
Height (cm)	160.1	149–182
Weight (kg)	60.3	43–79
BMI (kg/m^2)	23.5	18.3–31

### 2.4 Statistical Analysis of Perfusion Changes Induced by FIR Exposure

**FIGURE 4:**
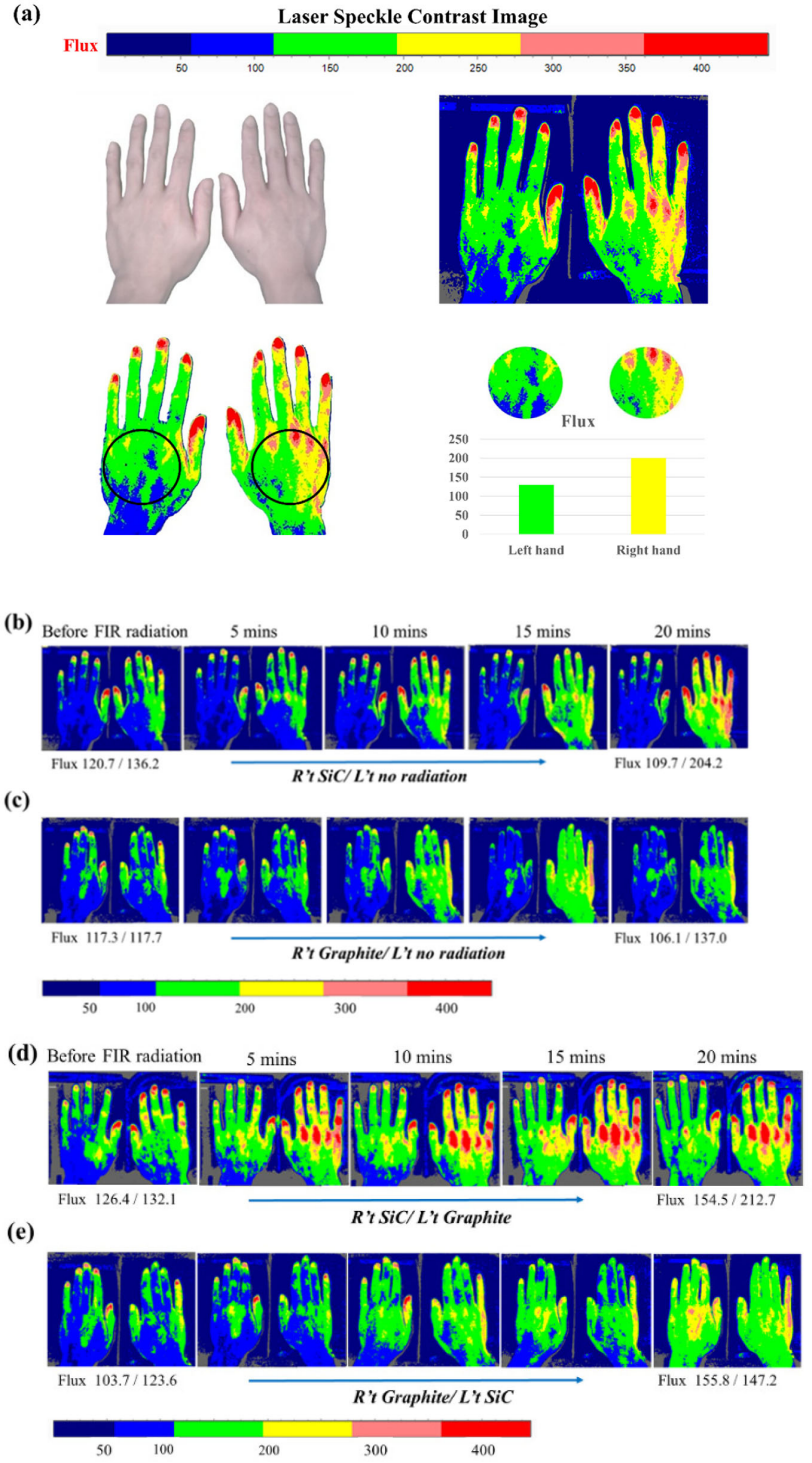
Blood flow assessment using laser speckle contrast imaging (LSCI) under different FIR irradiation conditions. (a) Representative example of LSCI analysis. The scale bar indicates relative blood flow (flux), and the measurement region was defined as a circular area over the dorsal hand. (b) The experimental condition where the right hand was irradiated using SiC, while the left hand received no irradiation and served as the control. (c) The condition where the right hand was irradiated using graphite, with the left hand serving as the non‐irradiated control. (d) Bilateral FIR exposure with the right hand irradiated by SiC and the left hand by graphite. (e) Reversed emitter placement, with the right hand irradiated by graphite and the left hand by SiC. All FIR emitters were mounted on hotplates and maintained at identical surface temperatures across all cases.

Flux values derived from LSCI were used to visualize cutaneous perfusion, displayed on a pseudocolor scale from blue (low) to red (high). Quantitative analysis was conducted over a circular region of interest (ROI, 6 cm diameter) on the dorsal hand (Figure 4a). A representative case is shown in Figure 4b, where the right hand irradiated with the SiC emitter showed an increase in perfusion from 136.2 to 204.2 Flux units (ΔFlux = +68.0) after 20 min. In contrast, the contralateral left hand, serving as the non‐irradiated control, exhibited a slight decrease from 120.7 to 109.7 (ΔFlux = –11.0). Similarly, graphite was used as an emitter on the right hand, and no radiation was applied to the left hand (Figure 4c). The graphite‐exposed hand exhibited an increase from 117.7 to 137.0 Flux units (ΔFlux = +19.3), whereas the non‐irradiated hand decreased from 117.3 to 106.1 (ΔFlux = −11.2).

In another representative case (Figure 4d), the right hand was exposed to the SiC emitter, while the left hand was exposed to the graphite emitter. After irradiation, the SiC‐exposed hand showed an increase in perfusion from 132.1 to 212.7 (ΔFlux = +80.6), whereas the graphite‐exposed hand increased more modestly from 126.4 to 154.5 (ΔFlux = +28.1). To eliminate positional bias, the emitter placement was alternated between the two hands in subsequent trials. When the emitter assignment was reversed (Figure 4e), the left hand irradiated with SiC showed an increase from 128.6 to 180.7 (ΔFlux = +52.1), while the right hand under graphite exposure increased from 121.5 to 145.1 (ΔFlux = +23.6). The initial baseline perfusion was slightly higher in the right hand, consistent with the participants’ dominant‐hand usage. These examples demonstrate how LSCI effectively captured perfusion dynamics in individual participants under different irradiation conditions, providing case‐level validation before group‐level statistical analysis.

**FIGURE 5:**
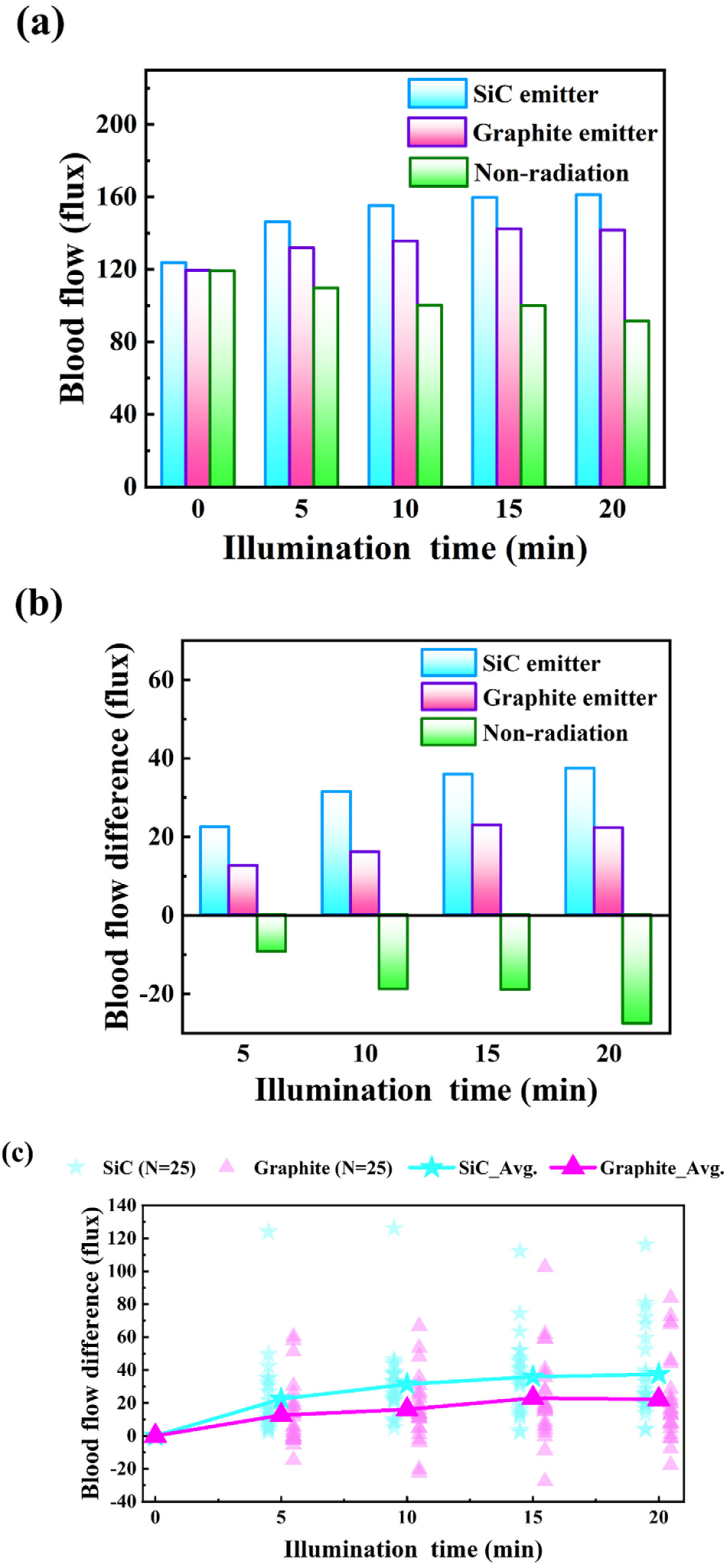
Quantitative analysis of blood flow changes under FIR irradiation with SiC and graphite emitters. (a) Comparative evaluation of blood flow enhancement after 20 min of illumination. The bar graph shows the flux between baseline and post‐irradiation levels for each material: cyan bars denote the SiC emitter, magenta bars denote the graphite emitter, and green bars represent the non‐radiation control. (b) Comparative analysis of blood flow difference (ΔFlux) after 20 min of irradiation. The bar graph illustrates ΔFlux at 5, 10, 15, and 20 min of illumination. Cyan, magenta, and green bars represent the SiC emitter, the graphite emitter, and the non‐radiation control, respectively. (c) Scatter plot summarizing the temporal progression of perfusion in the SiC and graphite emitters over 20 min of FIR exposure. The data reveal a consistently greater perfusion increase associated with the SiC emitter.

Case‐level imaging (Figure 4) revealed perfusion changes in individual participants. To extend these findings, all 25 volunteers (50 hands) were analyzed, and the group‐level outcomes are summarized in Figure 5. The temporal evolution of mean perfusion across the SiC (*n* = 25), graphite (*n* = 25), and non‐radiation control (*n* = 25) groups is depicted in Figure 5a. In the SiC group, baseline perfusion of 123.8 Flux units steadily increased to 146.2, 155.2, 159.7, and 161.2 Flux units at 5, 10, 15, and 20 min, respectively. Graphite exposure also enhanced perfusion, though to a lesser degree, increasing from 119.5 Flux units at baseline to 141.7 Flux units at 20 min. By contrast, the control group showed a gradual decline from 119.2 to 91.5 Flux units, reflecting the absence of thermal stimulation. These trajectories demonstrate that both emitters enhanced circulation relative to non‐irradiated controls, with SiC consistently producing the largest and most significant improvement.

To quantify relative changes, perfusion differences (ΔFlux) were calculated with respect to the baseline (Figure 5b). The SiC group showed a steady increase in ΔFlux, reaching +22.4, +31.4, +35.9, and +37.4 at successive 5‐minute intervals. The graphite group displayed more modest increases of +12.6, +16.1, +22.9, and +22.2 over the same period. In contrast, the non‐radiation control group showed declines of ΔFlux values of –9.4, –18.9, –19.1, and –27.7. These trends indicate that both selective‐band (SiC) and broadband (graphite) FIR emitters enhance peripheral blood flow under controlled thermal conditions. Furthermore, SiC produced a more pronounced and consistent effect, likely attributable to its spectral alignment with the 10 µm skin transparency window. Figure 5c shows individual responses, where SiC‐treated hands exhibited higher and more consistent ΔFlux values than those treated with graphite. Several graphite trials approached baseline or even fell below zero, highlighting greater variability in broadband emission.

Statistical validation is summarized in Table 2. Wilcoxon signed‐rank tests confirmed that the SiC group achieved significantly greater perfusion enhancement than the graphite group (*p* < 0.05) after 10 min of illumination. These results demonstrate that selective FIR irradiation using SiC is not only reproducible across a cohort of 25 participants but also statistically superior to broadband graphite under identical thermal conditions.

**TABLE 2:** Comparison of blood flow change (ΔFlux) over SiC and graphite.^a^

Illumination time (min)	SiC (*n* = 25) Mean± SD (Flux)	(ΔFlux)	graphite (*n* = 25) Mean± SD (Flux)	(ΔFlux)	*p*
0	123.8 ± 30.1	0	119.5 ± 20.1	0	—
5	146.2 ± 45.1	22.4	132.0 ± 30.1	12.6	0.059
10	155.2 ± 45.5	31.4	135.6 ± 30.4	16.1	0.006^b^
15	159.7 ± 46.2	35.9	142.4 ± 33.9	22.9	0.048^b^
20	161.2 ± 45.6	37.4	141.7 ± 29.6	22.2	0.011^b^

^a^ Wilcoxon signed‐rank test.

^b^ *p*<0.05 represents statistically significant.

### 2.5 Physiological Safety and Thermal Effects of FIR Irradiation

Physiological safety parameters were evaluated before and after FIR irradiation, as summarized in Table 3. No significant changes were observed in blood pressure (125/68 mmHg pre‐irradiation vs. 123/68 mmHg post‐irradiation, p = 0.237) or heart rate (75.8 vs. 74.4 beats per minute, p = 0.077). Oxygen saturation increased slightly from 97.0% to 97.7%; the change was not statistically significant (p = 0.064). In contrast, hand skin temperature increased significantly from 31.1°C to 34.0°C (*p* < 0.01) following FIR exposure. These findings confirm that the irradiation protocol was thermally safe and did not induce measurable alterations in systemic cardiovascular function. Targeted enhancement of peripheral circulation may improve local tissue oxygenation and nutrient delivery, thereby promoting wound healing.

**TABLE 3:** Comparison of vital signs before and after FIR treatment.^a^

Parameter (*n* = 25)	Before FIR treatment	After FIR treatment	*P*
Blood pressure (mmHg)	125/68	123/68	0.237
Heart rate (1/min)	75.8	74.4	0.077
Oxygen saturation (%)	97.0	97.7	0.064
Hand temperature (°C)	31.1	34.0	<0.01^b^

^a^ Wilcoxon signed‐rank test.

^b^ *P*<0.05 represents statistically significant.

In summary, the observed increase in cutaneous blood flow induced by SiC emitters, in the absence of concurrent physiological changes, indicates a localized microvascular response driven by spectral alignment rather than generalized thermal effects. This mechanism distinguishes SiC from conventional broadband FIR sources and underscores its potential for efficient and selective thermal modulation under controlled conditions.

## 3 Conclusion

Although FIR therapy has been extensively investigated, its clinical utility remains limited by the indiscriminate delivery of thermal energy from conventional broadband emitters and sauna‐based approaches. Human skin, however, exhibits an optical transmission window near 10 µm, where absorption is minimal, presenting an opportunity for more efficient thermal energy deposition. This study explored this principle by employing SiC, an ENZ material whose emission characteristics coincide with the ∼10 µm skin window, as a tailored FIR emitter. Spectroscopic analysis verified the alignment of SiC emission with the ∼10 µm skin window, providing a physical basis for enhanced thermal interaction. Building on this foundation, a thermally controlled within‐subject human study was conducted to compare SiC with broadband graphite under identical experimental conditions. A total of twenty‐five healthy volunteers, contributing 50 hands, were enrolled. Blood perfusion was monitored in real time using laser speckle contrast imaging, while systemic physiological parameters were simultaneously recorded to ensure safety.

The results demonstrated that SiC irradiation consistently enhanced cutaneous blood flow more effectively than broadband graphite emission. After 20 min, the SiC group exhibited an average 30.2% increase in perfusion flux relative to baseline, compared with 18.6% for graphite, with statistical analysis confirming significance at all time points. These enhancements occurred without measurable changes in blood pressure, heart rate, or oxygen saturation, while skin temperature remained below 36°C. The response reflects a localized microvascular effect rather than a generalized cardiovascular response. Overall, this study validates a mechanism‐driven approach to FIR therapy, showing that emission aligned with the intrinsic transmission properties of skin can achieve efficient circulatory modulation with minimal thermal load. Future studies should extend this strategy to patient populations with impaired microcirculation, investigate long‐term therapeutic efficacy, and elucidate the molecular mechanisms underlying FIR‐induced vasodilation. By establishing both mechanistic rationale and human evidence, this study provides a foundation for advancing FIR therapy toward precision, mechanism‐guided clinical applications.

## 4 Experimental Section/Methods

### 4.1 Sample Characteristics

The 4H‐SiC substrate had a diameter of 100 mm, a thickness of 500 µm, and an electrical resistivity of ≥ 1 × 10^8^ Ω·cm. WinSheng Material Technology Co., Ltd. was the supplier. The graphite thin film measured 150 × 150 mm, was 190 µm thick, and had a thermal conductivity of 20 W/m·K. It was obtained from T‐Global Technology Co., Ltd.

### 4.2 IR Optical Characterization

IR spectra of SiC and graphite were acquired using a VERTEX 70 FTIR (Bruker Optics Inc., Billerica, MA, USA). Human skin absorption spectra were measured under identical conditions using attenuated total reflectance FTIR (ATR‐FTIR). These measurements identified the low‐absorption FIR window of human skin and allowed direct comparison with the emission spectra of the selected emitter materials.

### 4.3 Blood Flow Measurement Setup

Blood flow was monitored using a FLPI‐2 laser speckle contrast imaging system (Moor Instruments, Axminster, UK). The manufacturer‐specified resolution of 3.9 µm corresponds to the theoretical pixel resolution at the minimum field of view. Under our experimental setup (working distance of 25 cm), the effective spatial resolution was calibrated to be approximately 233–234 µm per pixel based on image‐based measurements, as detailed in Table S6.

During experiments, emitter samples were mounted on a thermostatically controlled hotplate (model HP‐205; DJ Power, Taiwan) with a temperature range of 25°C–350°C. The emitters were positioned 5 cm above the dorsal surface of the hand. Skin surface temperature was continuously monitored using thermocouples to ensure values remained below 36°C. Blood flow dynamics were recorded in real time with a temporal resolution of 50 ms.

The study was conducted at MacKay Memorial Hospital (Taipei, Taiwan) and approved by the hospital's Institutional Review Board (IRB No. 24MMHIS205e). Written informed consent was obtained from all participants in accordance with the Declaration of Helsinki (1964) and its subsequent amendments or equivalent ethical standards.

### 4.4 Statistical Analysis

Data were analyzed using the Wilcoxon signed‐rank test, a non‐parametric method that does not assume normal distribution. Paired differences equal to zero were excluded. The absolute differences were ranked in ascending order, and average ranks were assigned in the presence of ties. The ranks were then grouped by sign and summed to obtain the signed‐rank statistic. The statistics were standardized, and the p‐value was calculated based on the normal distribution as follows:

(1) $$p=2\left(1-\Phi \left(\left|\frac{W-\frac{n\left(n+1\right)}{4}}{\sqrt{\frac{n\left(n+1\right)\left(2n+1\right)}{24}}}\right|\right)\right)$$

where W is the sum of signed ranks, n is the number of non‐zero paired differences, and Φ denotes the cumulative distribution function of the standard normal distribution. A two‐tailed test was applied, and statistical significance was defined as *p* < 0.05.

## Author Contributions

W.‐T. Y. and S.‐C. Y. contributed equally to this work. W.‐T. Y. and S.‐C. Y. prepared the manuscript. W.‐T. Y. conducted the experiments with supportfrom S.‐C. Y., and M.‐F. T. assisted with statistical analysis. M.‐F. T. helped the Institutional Review Board (IRB) application. S.‐C. Y. and H.‐L. C. offered guidance on the experimental setup. H.‐L. C. contributed conceptual ideas. All authors participated in data analysis and manuscript development.

## Conflicts of Interest

The authors declare no conflicts of interest.

## Supporting information




**Supporting File**: advs75938‐sup‐0001‐SuppMat.docx.

## Data Availability

The data that support the findings of this study are available on request from the corresponding author. The data are not publicly available due to privacy or ethical restrictions.
