# Supplementary material for: Enhancing Blood Circulation With Epsilon‐Near‐Zero (ENZ) Materials via the Far‐Infrared Window of Human Skin
Source: Adv Sci (Weinh). 2026 Jun 15:e75938. Online ahead of print. doi: 10.1002/advs.75938 (PMC13336465; doi:10.1002/advs.75938)
Supplement: Supplementary file 1 — Supporting File: advs75938‐sup‐0001‐SuppMat.docx. [file ADVS-9999-e75938-s001.docx]

Supporting information for

**Enhancing Blood Circulation with Epsilon-near-zero (ENZ) Materials via the Far-Infrared Window of Human Skin**

*Wen-Teng Yao^1, 2, 3^, Shan-Chiao Yang^1^, Ming-Feng Tsai^2, 3^, and Hsuen-Li Chen^1, 4 *^*

^1^ Department of Materials Science and Engineering, National Taiwan University, No. 1, Section 4, Roosevelt Road, Taipei, 10617, Taiwan

^2^Department of Medicine, MacKay Medical University, No.46, Sec. 3, Zhongzheng Rd., Sanzhi Dist., New Taipei City, Taiwan

^3^Division of Plastic Surgery, Department of Surgery, Mackay Memorial Hospital, No. 92, Sec. 2, Zhongshan N. Rd., Zhongshan Dist., Taipei, Taiwan

^4^Center of Atomic Initiative for New Materials, National Taiwan University, No. 1, Section 4, Roosevelt Road, Taipei, 10617, Taiwan

^*^ Corresponding authors

E-mail: hsuenlichen@ntu.edu.tw; Tel: +886-3366-3240

**Supporting Information 1:**

**Table S1 SiC group: Blood flow (Flux) under 20 minutes illumination**

| flux  minutes | SiC | | | | |
| --- | --- | --- | --- | --- | --- |
|  | No.1 | No.2 | No.3 | No.4 | No.5 |
| 0 | 164.8 | 170.6 | 167.8 | 156 | 89.6 |
| 5 | 288.6 | 185.5 | 183.7 | 160.3 | 98.3 |
| 10 | 290.7 | 216.9 | 190.9 | 184.6 | 98.7 |
| 15 | 276.8 | 188.1 | 202.8 | 187.1 | 91.4 |
| 20 | 243.1 | 183.8 | 192.5 | 215.2 | 93.1 |
|  |  |  |  |  |  |
|  | No.6 | No.7 | No.8 | No.9 | No.10 |
| 0 | 139.4 | 148 | 136.2 | 63.2 | 154.1 |
| 5 | 181.4 | 153.9 | 168.2 | 73.6 | 184.3 |
| 10 | 159.9 | 178.2 | 168.5 | 73.1 | 191.7 |
| 15 | 186.7 | 163 | 187.8 | 66.2 | 187.5 |
| 20 | 161.3 | 167 | 204.2 | 83.5 | 182.3 |
|  |  |  |  |  |  |
|  | No.11 | No.12 | No.13 | No.14 | No.15 |
| 0 | 99.6 | 124.1 | 76.7 | 168.4 | 110.3 |
| 5 | 134.5 | 145.4 | 80.3 | 177.6 | 123.1 |
| 10 | 142.7 | 146 | 81.3 | 196.1 | 143.4 |
| 15 | 145.3 | 154.1 | 89.2 | 208.4 | 154.7 |
| 20 | 132.1 | 148.3 | 93 | 187.1 | 129.9 |
|  |  |  |  |  |  |
|  | No.16 | No.17 | No.18 | No.19 | No.20 |
| 0 | 132.1 | 101.3 | 103.7 | 139.5 | 119.6 |
| 5 | 181.4 | 103.3 | 130.2 | 174 | 126.7 |
| 10 | 168.8 | 123.8 | 148.1 | 164.9 | 152.1 |
| 15 | 206.3 | 112.2 | 136.6 | 202.6 | 133 |
| 20 | 212.7 | 105 | 155.8 | 255.4 | 158.9 |
|  |  |  |  |  |  |
|  | No.21 | No.22 | No.23 | No.24 | No.25 |
| 0 | 116.4 | 105.7 | 109.5 | 90.6 | 107.4 |
| 5 | 131.5 | 122.9 | 126.7 | 95.4 | 124.8 |
| 10 | 154.8 | 130.5 | 130.9 | 98.3 | 144.8 |
| 15 | 168.2 | 138.7 | 147 | 110 | 148.6 |
| 20 | 188.2 | 143.5 | 134.8 | 126.1 | 132.9 |

**Table S2: Graphite group: Blood flow (Flux) under 20 minutes illumination**

| flux  minutes | graphite | | | | |
| --- | --- | --- | --- | --- | --- |
|  | No.1 | No.2 | No.3 | No.4 | No.5 |
| 0 | 126.4 | 134.1 | 154.8 | 99.1 | 119 |
| 5 | 177.3 | 147.6 | 172.9 | 97.4 | 122.2 |
| 10 | 161.6 | 152.7 | 167.5 | 112.1 | 114.8 |
| 15 | 161.8 | 133.4 | 158.4 | 126.7 | 125.4 |
| 20 | 154.5 | 126 | 170.8 | 144.7 | 132 |
|  |  |  |  |  |  |
|  | No.6 | No.7 | No.8 | No.9 | No.10 |
| 0 | 130.9 | 66 | 120.7 | 109.6 | 123.6 |
| 5 | 146.8 | 86.1 | 120.2 | 103.9 | 120.8 |
| 10 | 144.4 | 83.5 | 100.1 | 86.8 | 128 |
| 15 | 139.2 | 85.2 | 122.3 | 100.6 | 141 |
| 20 | 138.4 | 78.3 | 121.3 | 91.6 | 147.2 |
|  |  |  |  |  |  |
|  | No.11 | No.12 | No.13 | No.14 | No.15 |
| 0 | 131.8 | 93.9 | 89.1 | 105.8 | 132.1 |
| 5 | 130.7 | 154 | 107.3 | 90.8 | 162.2 |
| 10 | 142.4 | 147.1 | 119.7 | 103.3 | 198.6 |
| 15 | 148.1 | 155.5 | 147.8 | 77.9 | 191.2 |
| 20 | 146.1 | 161.5 | 161.6 | 118.3 | 176 |
|  |  |  |  |  |  |
|  | No.16 | No.17 | No.18 | No.19 | No.20 |
| 0 | 151.6 | 123.6 | 131.8 | 93.1 | 117.7 |
| 5 | 209.3 | 120.8 | 130.7 | 90.3 | 129.1 |
| 10 | 199.5 | 128 | 142.4 | 93.8 | 144.2 |
| 15 | 254 | 141 | 148.1 | 119.9 | 157.9 |
| 20 | 235.2 | 130.2 | 130.1 | 161.9 | 137 |
|  |  |  |  |  |  |
|  | No.21 | No.22 | No.23 | No.24 | No.25 |
| 0 | 126.1 | 111 | 129.4 | 126.2 | 139.3 |
| 5 | 131.4 | 112.5 | 131 | 140.1 | 165.3 |
| 10 | 151 | 122 | 145.1 | 139 | 162 |
| 15 | 163.7 | 129.2 | 145.5 | 131.6 | 153.8 |
| 20 | 130.3 | 132.5 | 137.2 | 124.1 | 155.5 |

**Table S3: Non-radiation group: Blood flow (Flux) with 20 minutes non-radiation**

| flux  minutes | Non-radiation | | | | |
| --- | --- | --- | --- | --- | --- |
|  | No.1 | No.2 | No.3 | No.4 | No.5 |
| 0 | 125.6 | 120.7 | 121.7 | 121.8 | 121.2 |
| 5 | 129.9 | 122.3 | 103.8 | 106.9 | 124.6 |
| 10 | 112.9 | 112.5 | 109 | 104.1 | 110.3 |
| 15 | 135.1 | 116.1 | 104.7 | 105.9 | 132.7 |
| 20 | 110.9 | 109.7 | 88.5 | 91.4 | 107.6 |
|  |  |  |  |  |  |
|  | No.6 | No.7 | No.8 | No.9 | No.10 |
| 0 | 117.3 | 103.1 | 114.7 | 94 | 106 |
| 5 | 119.4 | 125.4 | 110.7 | 82.1 | 93 |
| 10 | 108.7 | 114.6 | 109.5 | 80.7 | 86.6 |
| 15 | 124.4 | 112.4 | 116.7 | 83.7 | 88.1 |
| 20 | 106.1 | 128.9 | 116.4 | 81.2 | 75.9 |
|  |  |  |  |  |  |
|  | No.11 | No.12 | No.13 | No.14 | No.15 |
| 0 | 120.3 | 110.6 | 151.3 | 148.3 | 99.6 |
| 5 | 108.3 | 86.4 | 133 | 122.1 | 95.4 |
| 10 | 89.1 | 49.3 | 112.9 | 101.3 | 81 |
| 15 | 70.4 | 51.2 | 107.7 | 74.6 | 78 |
| 20 | 79.3 | 48.3 | 71.4 | 57.2 | 72.2 |
|  |  |  |  |  |  |
|  | No.16 | No.17 | No.18 | No.19 | No.20 |
| 0 | 109.6 | 106.5 | 121.8 | 128.1 | 104 |
| 5 | 99.3 | 84 | 118.5 | 124.2 | 104 |
| 10 | 85.2 | 80.6 | 108.2 | 118.6 | 102.7 |
| 15 | 94.9 | 77.6 | 111.2 | 113.4 | 95.5 |
| 20 | 87.4 | 81 | 113.6 | 103.1 | 80.2 |
|  |  |  |  |  |  |
|  | No.21 | No.22 | No.23 | No.24 | No.25 |
| 0 | 139.2 | 136.3 | 97.2 | 144.1 | 116.72 |
| 5 | 111.4 | 136.7 | 96.5 | 114.9 | 92.4 |
| 10 | 136.2 | 124.5 | 85.8 | 103.2 | 79.7 |
| 15 | 119.5 | 122.8 | 82.6 | 111.6 | 71 |
| 20 | 109.8 | 122.4 | 83.4 | 104.4 | 56.2 |

**Supporting Information 2:**

**Figure S1** Spectral irradiance of the SiC emitter at 383 K (cyan line) and blackbody radiation at different temperatures

**Supporting Information 3:**

In an isothermal control experiment, both hands were allowed to increase in skin temperature by approximately 3.5 °C using either SiC or broadband graphite emitters, thereby isolating thermal effects from spectral influences. Under these thermal conditions, the SiC group demonstrated a markedly higher increase in blood flow (35.1%) compared to the graphite group (19.3%), indicating that spectrally selective emission provides an additional enhancement beyond baseline thermoregulatory vasodilation. The detailed results are summarized in **Table S4**.e

**Table S4** **Isothermal control experiment**

|  | 0 min | 10 mins | 20 mins | Difference |
| --- | --- | --- | --- | --- |
| Temperature of right hand  (SiC) | 31.2 ^o^C | 32.1 ^o^C | 34.6 ^o^C | +3.4 ^o^C |
| Temperature of left hand  (graphite) | 31.0 ^o^C | 32.0 ^o^C | 34.5 ^o^C | +3.5 ^o^C |
| Flux of right hand  (SiC) | 88.7 | 111.8 | 119.8 | +31.1  (35.1%) |
| Flux of left hand  (graphite) | 89.5 | 95.2 | 106.8 | +17.3  (19.3%) |

**Supporting Information 4:**

**
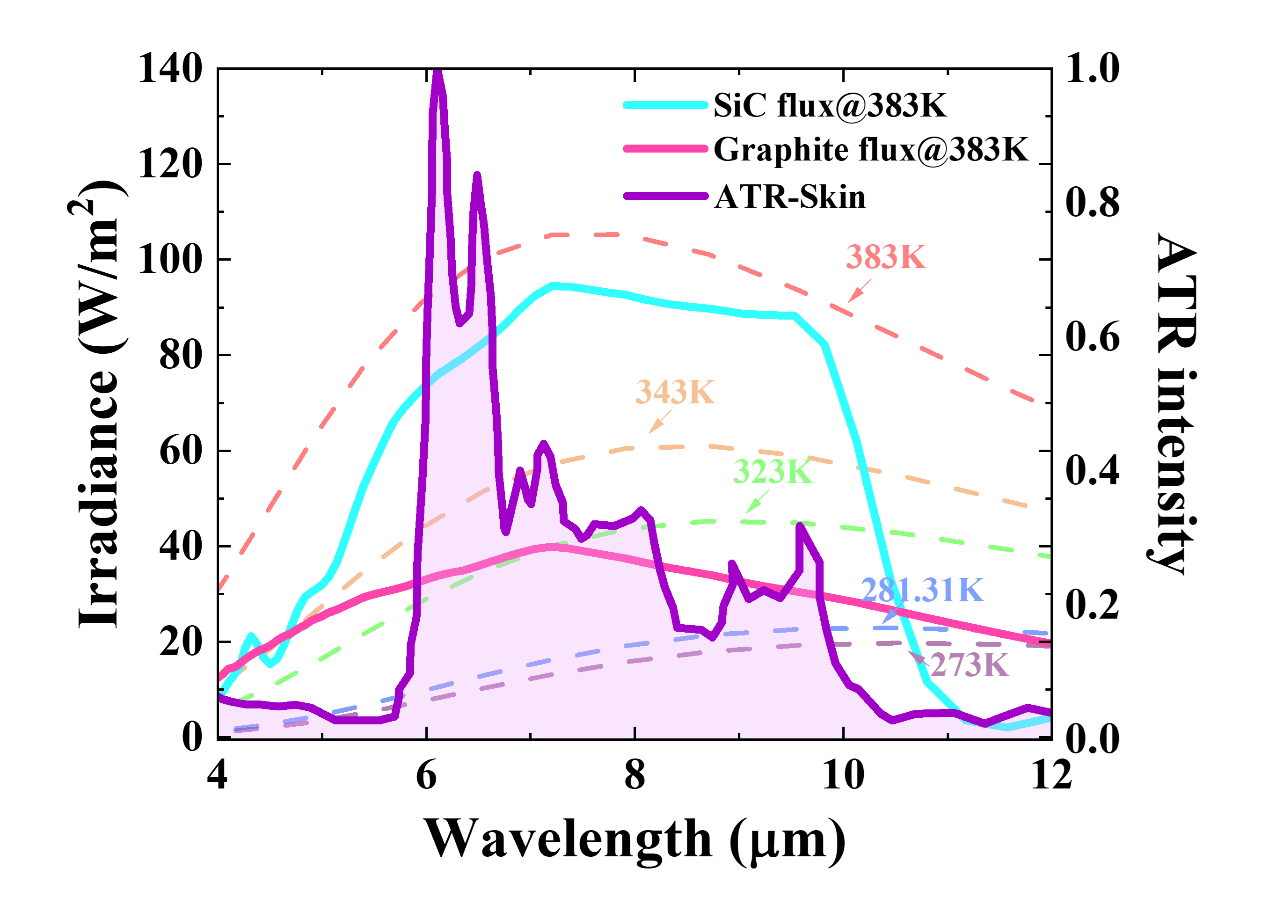
Figure S2** Weighted emissivity of SiC (cyan) and graphite (magenta) at 110 °C and overlaid with the ATR-FTIR spectrum of human skin

**Supporting Information 5:**


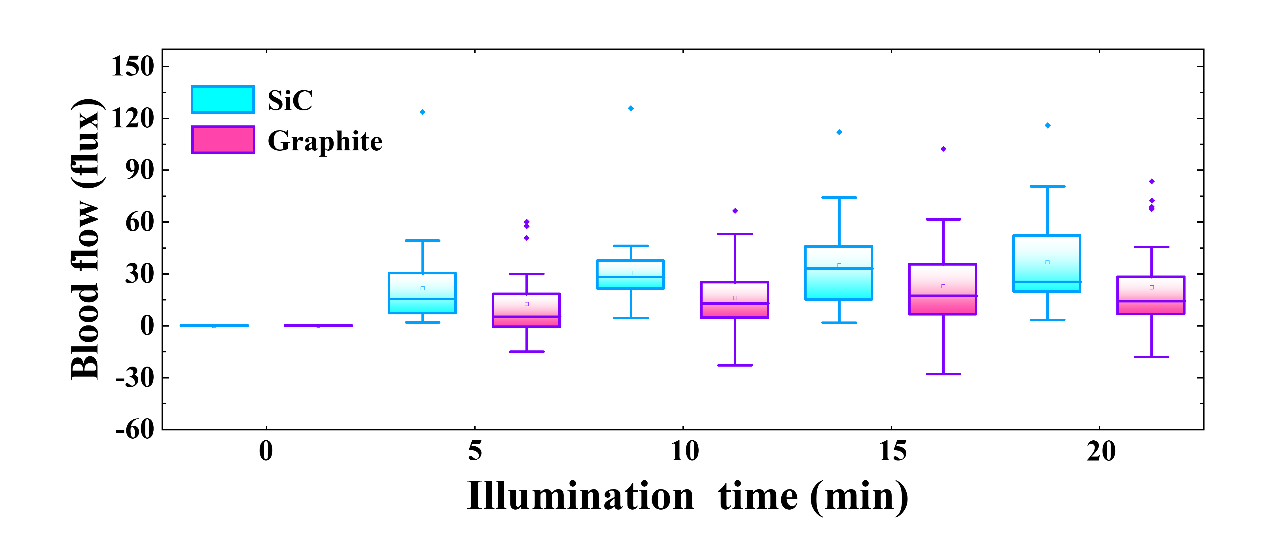
**Figure S3** Box plot of blood flow of SiC (cyan) and graphite (magenta)

**Supporting Information 6:**

In an additional neutral heat control experiment, we evaluated the effect of non-specific thermal stimulation by increasing hand temperature by approximately 3.6 °C, which resulted in a modest rise in blood flow from 84.3 to 95.1 (+12.8%). In contrast, under a comparable temperature increase of 3.7 °C using the SiC emitter, blood flow increased substantially from 90.6 to 126.1 (+39.2%). These findings indicate that general heating alone cannot account for the observed enhancement in blood flow, supporting an additional contribution from spectral selectivity. The corresponding data are provided in **Table S5**.

**Table S5** Neutral heat experiment

|  | 0 min | 5 mins | 10 mins | 15 mins | 20 mins | Difference |
| --- | --- | --- | --- | --- | --- | --- |
| Temperature of Neutral heat | 30.9°C | 31.7°C | 32.5°C | 33.4°C | 34.5°C | +3.6°C |
| Hand Flux of Neutral heat | 84.3 | 86.1 | 89.1 | 91.9 | 95.1 | +10.8  (12.8%) |
| Temperature of SiC | 31.2°C | 31.7°C | 32.5°C | 33.4°C | 34.9°C | +3.7°C |
| Hand Flux of SiC | 90.6 | 95.4 | 98.3 | 110.9 | 126.1 | +35.5  (39.2%) |

**Supporting Information 7:**

We also have supplemented the manuscript with measurements of the incident power density using a calibrated radiometer in combination with four bandpass filters, and the results are now presented in **Figures S4** and **S5**. The corresponding center transmission wavelengths of the filters, measured from FTIR characterization, are approximately 2.5 μm (Filter 1), 4.7 μm (Filter 2), 7.5 μm (Filter 3), and 10.6 μm (Filter 4). The measured band-specific and total incident power densities are shown in the figure.

Across all spectral bands and in total power density, the SiC emitter consistently exhibits a higher incident power density than graphite, confirming the effectiveness of the selective emitter in enhancing emission within the targeted wavelength regions.


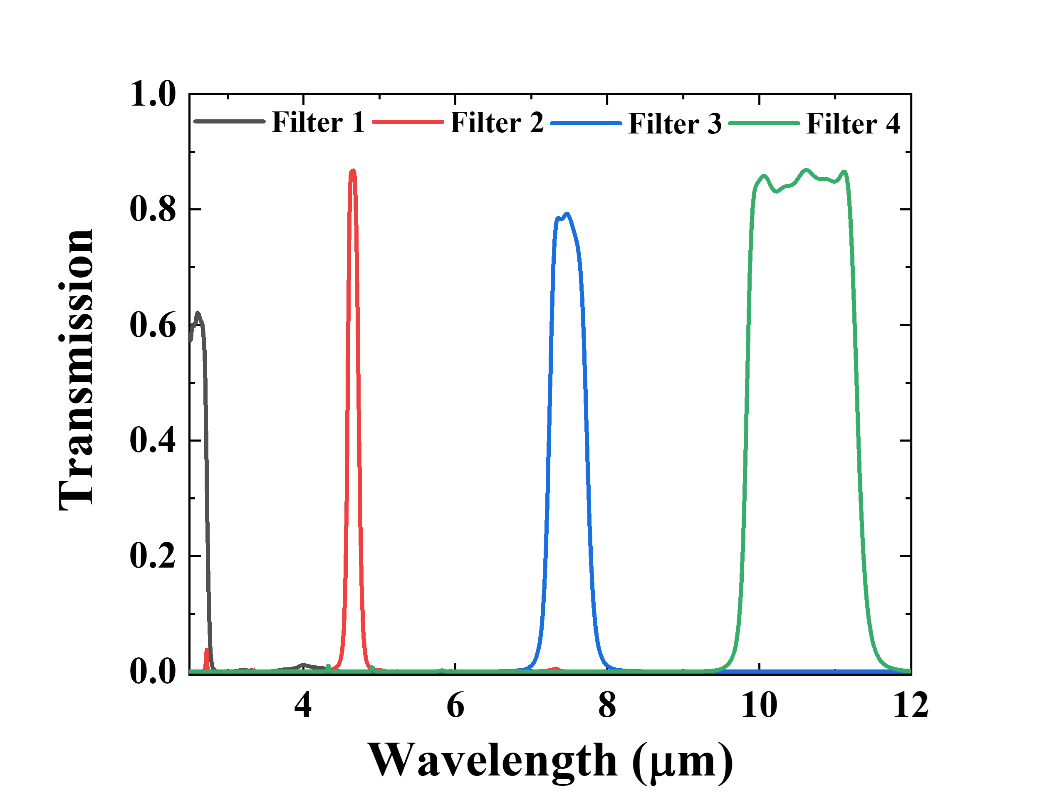


**Figure S4** Transmission spectrum of bandpass filters


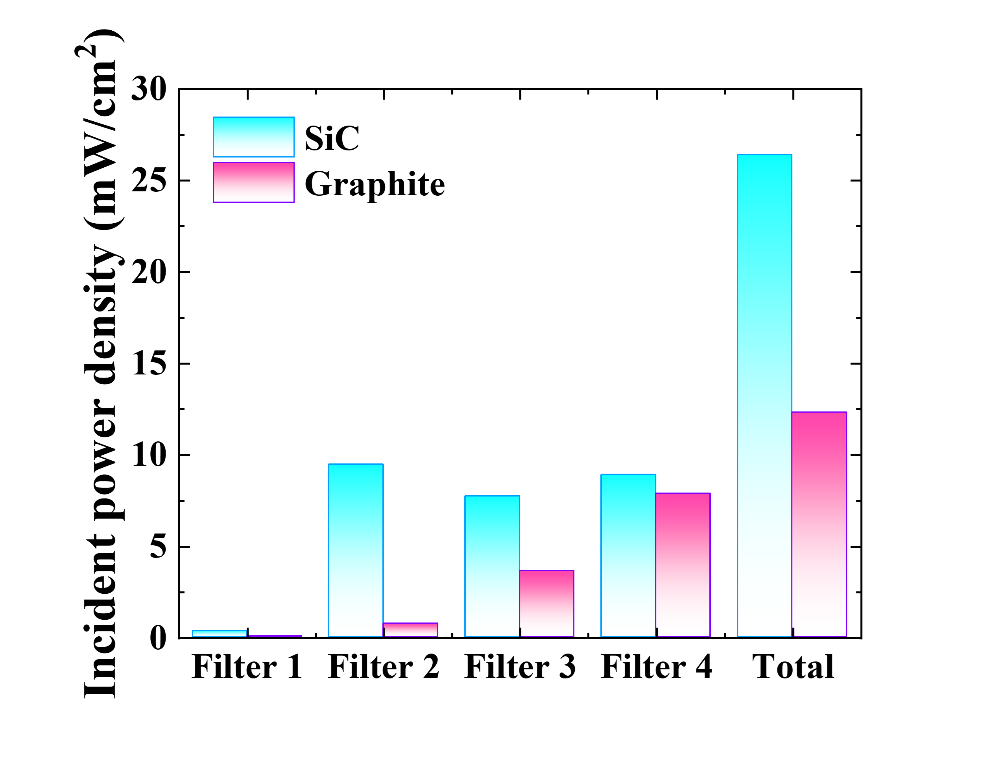


**Figure S5** Band-specific and total irradiance of the SiC and graphite emitters

**Supporting Information 8:**

**Table S6** LSCI resolution analysis

| Line length(mm) | Line pixels | Pixel Resolution (μm) |
| --- | --- | --- |
| 35 | 150 | 0.233 |
| 100 | 429 | 0.233 |
| 50 | 214 | 0.234 |
| 22 | 94 | 0.234 |
| 145 | 622 | 0.233 |
